# Machine learning-based radiomics using MRI to differentiate early-stage Duchenne and Becker muscular dystrophy in children

**DOI:** 10.1186/s12891-025-08538-7

**Published:** 2025-03-22

**Authors:** Taiya Chen, Haoran Zhu, Yingyi Hu, Yang Huang, Wengan He, Yizhen Luo, Zeqi Wu, Diangang Fang, Longwei Sun, Hongwu Zeng, Zhiyong Li

**Affiliations:** 1 Department of Radiology, Shenzhen People’s Hospital, The Second Clinical Medical College, Jinan University, Shenzhen, China; 2 Department of Radiology, Shenzhen Children’s Hospital, Shantou University Medical College, Shenzhen, China; 3 School of Biomedical Engineering, Shenzhen University Medical School, Shenzhen University, Shenzhen, China; 4 Department of Radiology, Shenzhen Children’s Hospital, China Medical University, Shenzhen, China; 5 Shenzhen ZhenData Intelligent Technology Co., Ltd, Shenzhen, China

**Keywords:** Duchenne muscular dystrophy, Becker muscular dystrophy, Muscle MRI, Early diagnosis, Radiomics, Machine learning

## Abstract

**Objectives:**

Duchenne muscular dystrophy (DMD) and Becker muscular dystrophy (BMD) present similar symptoms in the early stage, complicating their differentiation. This study aims to develop a classification model using radiomic features from MRI T2-weighted Dixon sequences to increase the accuracy of distinguishing DMD and BMD in the early disease stage.

**Methods:**

We retrospectively analysed MRI data from 62 patients aged 36–60 months with muscular dystrophy, including 41 with DMD and 21 with BMD. Radiomic features were extracted from in-phase, opposed-phase, water, fat, and postprocessed fat fraction images. We employed a deep learning segmentation method to segment regions of interest automatically. Feature selection included the Mann‒Whitney U test for identifying significant features, Pearson correlation analysis to remove collinear features, and the LASSO regression method to select features with nonzero coefficients. These selected features were then used in various machine learning algorithms to construct the classification model, and their diagnostic performance was compared.

**Results:**

Our proposed radiomic and machine learning methods effectively distinguished early DMD and BMD. The machine learning models significantly outperformed the radiologists in terms of accuracy (81.2-90.6% compared with 69.4%), specificity (71.0-86.0% compared with 19.0%), and F1 score (85.2-92.6% compared with 80.5%), while maintaining relatively high sensitivity (85.6-95.0% compared with 95.1%).

**Conclusion:**

Radiomics based on Dixon sequences combined with machine learning methods can effectively distinguish between DMD and BMD in the early stages, providing a new and effective tool for the early diagnosis of these muscular dystrophies.

**Clinical trial number:**

Not applicable.

**Supplementary Information:**

The online version contains supplementary material available at 10.1186/s12891-025-08538-7.

## Introduction

Muscular dystrophy (MD) is a group of genetic, progressive muscle degenerative disorders that are characterized primarily by gradual muscle atrophy and strength decline [1]. These conditions commonly impair motor functions and, in severe instances, can precipitate respiratory and cardiac failure [2]. MD can be categorized into various subtypes on the basis of genetic background and clinical manifestations [3]. Specifically, Duchenne muscular dystrophy (DMD) and Becker muscular dystrophy (BMD) are the most common and severe subtypes of MD [4–6]. DMD typically manifests in early childhood and is characterized by symptoms such as difficulty walking and frequent falls. In DMD, muscle weakness typically begins in the proximal muscles of the lower limbs, such as the gluteus maximus and adductor muscles, and progresses to the upper limbs and other muscle groups. As the disease progresses, patients often require wheelchair support by adolescence and may experience life-threatening respiratory failure or cardiac complications in their twenties [7, 8]. In contrast, BMD progresses more slowly with symptoms similar to those of DMD. While similar muscle groups are affected, the severity is generally milder. Many patients with BMD retain a considerable degree of physical activity into adulthood, and their life expectancy is generally extended [9]. Clinical studies have demonstrated that early and sustained use of corticosteroids can significantly prolong the period of independent walking in patients with DMD, improve their cardiopulmonary functions, and enhance overall quality of life, thereby increasing survival [39]. Currently, the international guidelines suggest the use of daily corticosteroids as standard treatment and the implementation of multidisciplinary care for patients with DMD who have completed basic immunization but relatively loose strategies for patients with BMD [13]. Given the side effects associated with long-term use of glucocorticoids and the potential therapeutic benefits, the advisability of their routine or prolonged use in patients with BMD remains to be verified [40]. Therefore, precise identification and early diagnosis of dystrophinopathies are crucial to maximize treatment benefits for DMD patients and minimize the risk of overtreatment in those with BMD.

The diagnosis and differentiation of BMD and DMD primarily rely on the assessment of clinical symptoms, muscle biopsy, genetic testing, and muscle MRI [8]. The above methods can provide evidence for the differentiation of BMD from DMD. The ‘Reading-Frame rule’ is the most commonly used method to differentiate these conditions, but it is not applicable to all patients [32, 33]. Next-generation sequencing (NGS) has become a widely used tool for diagnosing neuromuscular diseases and efficiently identifies genetic mutations associated with conditions such as muscular dystrophy [42]. However, its ability to differentiate between the subtypes of DMD and BMD still requires further investigation. While muscle biopsy provides valuable diagnostic information, it is an invasive procedure and may also cause collateral tissue damage. Clinical symptoms often fail to provide clear evidence in the early stages of the disease [35, 36]. Specifically, muscle MRI via Dixon water‒fat separation techniques reveals signs of muscle edema, atrophy, and fatty replacement, offering a noninvasive and cost-effective alternative to other diagnostic approaches [10, 11]. However, in the early stages of both conditions, similar patterns of fat infiltration in muscle tissue frequently occur, thus complicating the diagnostic process [4, 12]. Early and accurate differentiation of these two diseases through MRI can provide critical information for timely medical intervention, which may considerably enhance patient quality of life and improve prognostic outcomes [13–15].

**Fig. 1:**
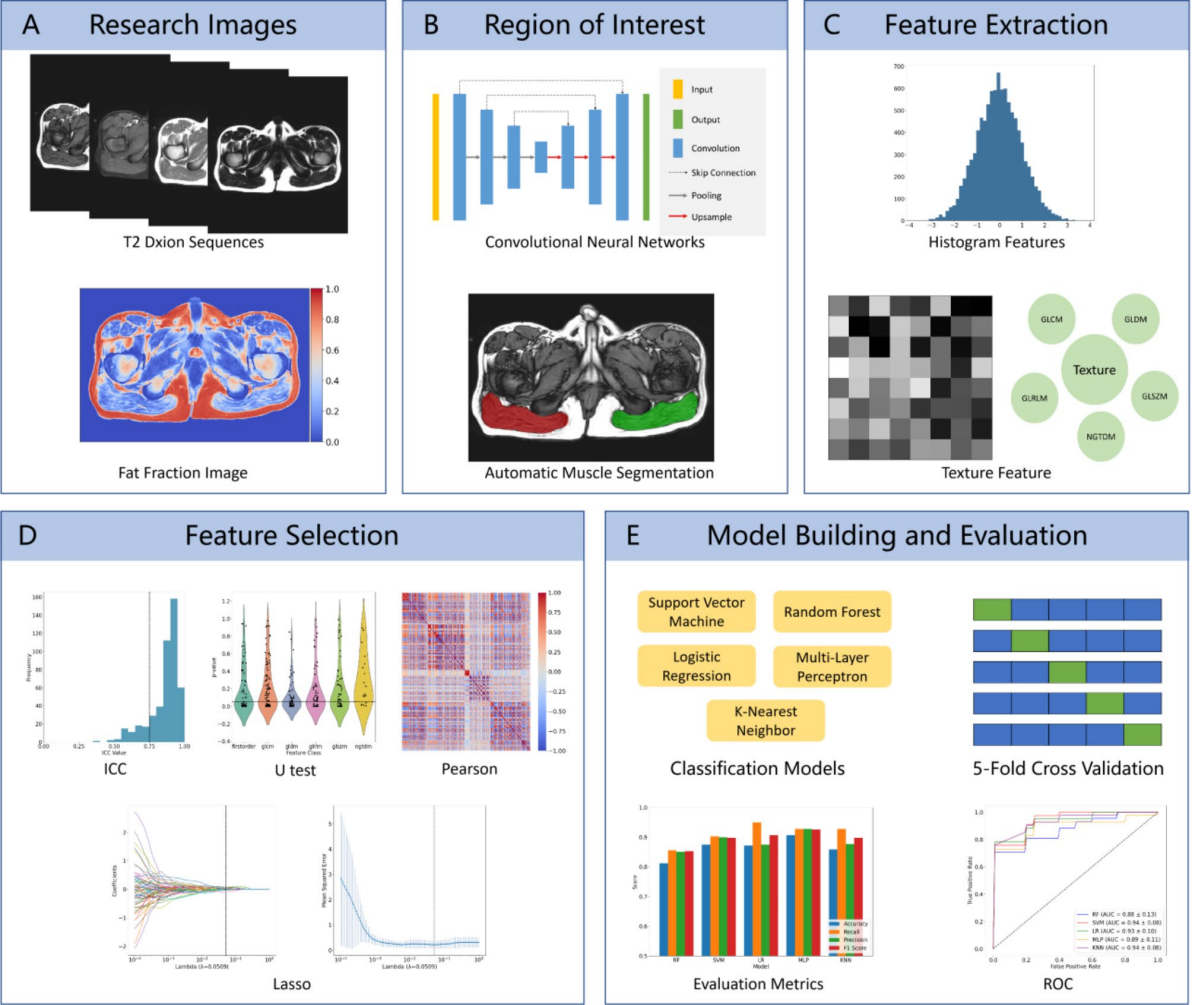
Radiomics modeling and analysis workflow

Radiomics and machine learning (ML) technologies provide new perspectives for medical data analysis [16]. Radiomics enables the extraction of numerous quantitative features from medical images, which provide essential data for further research. ML algorithms can build models from these data, which can then be used for disease classification or progression prediction [17, 18]. This study integrates radiomics and ML to analyse thigh Dixon sequence images of patients, aiming to accurately differentiate between DMD and BMD in the early stages of the disease (36–60 months). The application of this methodology is expected to provide more reference information for early clinical classification and facilitate the development of personalized treatment plans. The workflow of the study is depicted in Fig. 1.

## Materials and methods

### Study population

This study was retrospective and received approval from the Institutional Review Board of Shenzhen Children’s Hospital. Between 2014-12-22 and 2020-6-14, a total of 109 male patients suspected of having DMD or BMD were admitted to our institution. The inclusion criteria included patients who had undergone lower limb muscle MRI T2-weighted Dixon sequence scans at our institution and were suspected of having DMD or BMD. The exclusion criteria were as follows: (a) age outside the 36–60 month range at the time of MRI; (b) lack of sufficient diagnostic evidence for DMD or BMD as of May 2024; and (c) incomplete or unqualified MRI sequences.

After screening, 62 patients were diagnosed with DMD or BMD through a combination of clinical symptoms, MRI, genetic testing, muscle biopsy, or electromyography. Ultimately, 41 patients diagnosed with DMD and 21 patients diagnosed with BMD were included in this study.

### MRI acquisition

All MRI examinations were conducted via a 3.0 Tesla Siemens Skyra scanner (Siemens Medical Solutions, Erlangen, Germany). All patients were sedated with 10% chloral hydrate enema (0.5 ml/kg, Qingdao, China) before the MRI scan because of their inability to cooperate, and all of them were placed in the supine position with their legs spread. The scanning area was from the anterior superior iliac spine to the distal femur. The scanning parameters were as follows: Axial 2-point T2WI-DIXON: TR/TE1/TE2 3500 ms/1.72 ms/2.74 ms; slice thickness, 6 mm; slice interval, 1.2 mm; FOV, 250 mm×150 mm; acquisition time, 2 min; and flip angle, 150°.

**Fig. 2:**
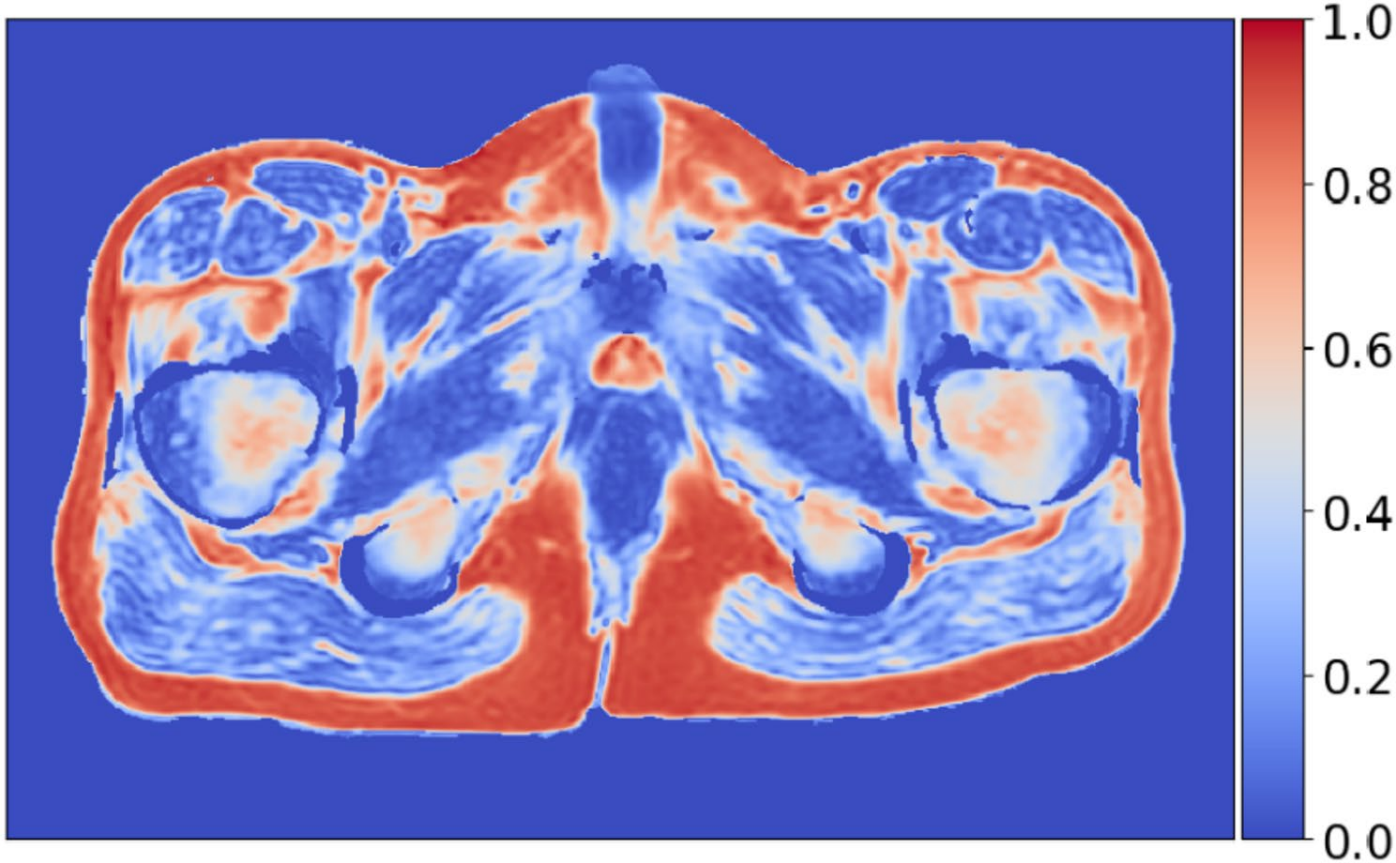
Fat fraction image

Fat fraction images were derived from the water and fat images obtained through the Dixon sequence via the following formula:

$$\:\varvec{F}\varvec{a}\varvec{t}\:\varvec{F}\varvec{r}\varvec{a}\varvec{c}\varvec{t}\varvec{i}\varvec{o}\varvec{n}=\frac{{\varvec{S}}_{\varvec{f}\varvec{a}\varvec{t}}}{{\varvec{S}}_{\varvec{f}\varvec{a}\varvec{t}}+{\varvec{S}}_{\varvec{w}\varvec{a}\varvec{t}\varvec{e}\varvec{r}}}$$

where $\:{S}_{fat}$ and $\:{S}_{water}$ represent the signal intensities of the fat and water sequences, respectively. The resulting fat fraction map is visualized as a heatmap, as shown in Fig. 2.

### Clinical assessment

Usually, the gluteus maximus is one of the first muscles affected by fatty infiltration in muscular dystrophy [2]. However, DMD and BMD show highly similar characteristics on MRI scans in the early stages of disease progression, which significantly complicates the challenge of clinical diagnosis [4]. As a result, few studies have explored the differences in the gluteus maximus between BMD patients and DMD patients during the early phase via MRI. To demonstrate the effectiveness of our proposed method, we implemented the following procedure to evaluate the performance of clinical diagnostics:

Two experienced radiologists (with 17 years and 5 years of experience in muscular radiology, both with substantial experience in differentiating DMD and BMD from MRI) independently assessed the T2-weighted Dixon MRI sequences of all patients. Each radiologist was provided with a standardized set of MR images, where the patients were diagnosed with either DMD or BMD. The radiologists were tasked with distinguishing whether the images corresponded to DMD or BMD patients, were blinded to the patient’s clinical histories and lacked knowledge of each other’s evaluations. The radiologist reviewed the images and recorded the Mercuri and edema scores of each patient’s gluteus maximus, along with the final diagnostic outcomes [19, 38]. Specifically, the Mercuri score is used to assess the extent of muscle fat replacement, and the edema score is used to quantify muscle edema. In cases of diagnostic disagreement, the radiologists discuss with each other, ensuring the accuracy and consistency of the diagnoses. The results of these evaluations are compared with the results derived from the method proposed in this study.

### Region of interest segmentation

**Fig. 3:**
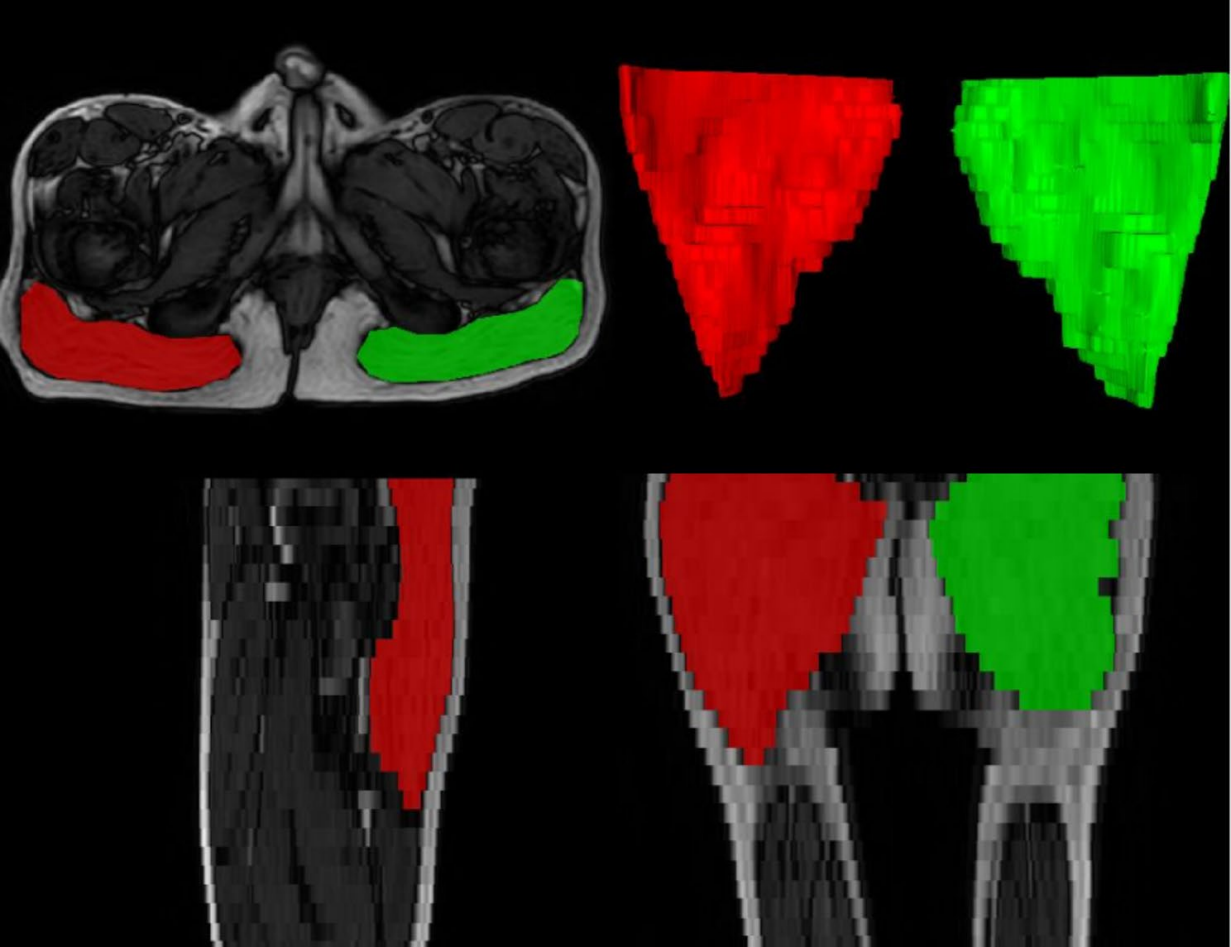
Annotated image of the gluteus maximus

In this work, we selected the bilateral gluteus maximus as the focal region. The segmentation of the muscle area was completed by a radiologist via the semiautomatic annotation tool iMOS [20]. To ensure the accuracy and consistency of the segmentation, the results were reviewed and adjusted by another radiologist. An example of bilateral gluteus maximus segmentation and its 3D display is shown in Fig. 3.

**Fig. 4:**
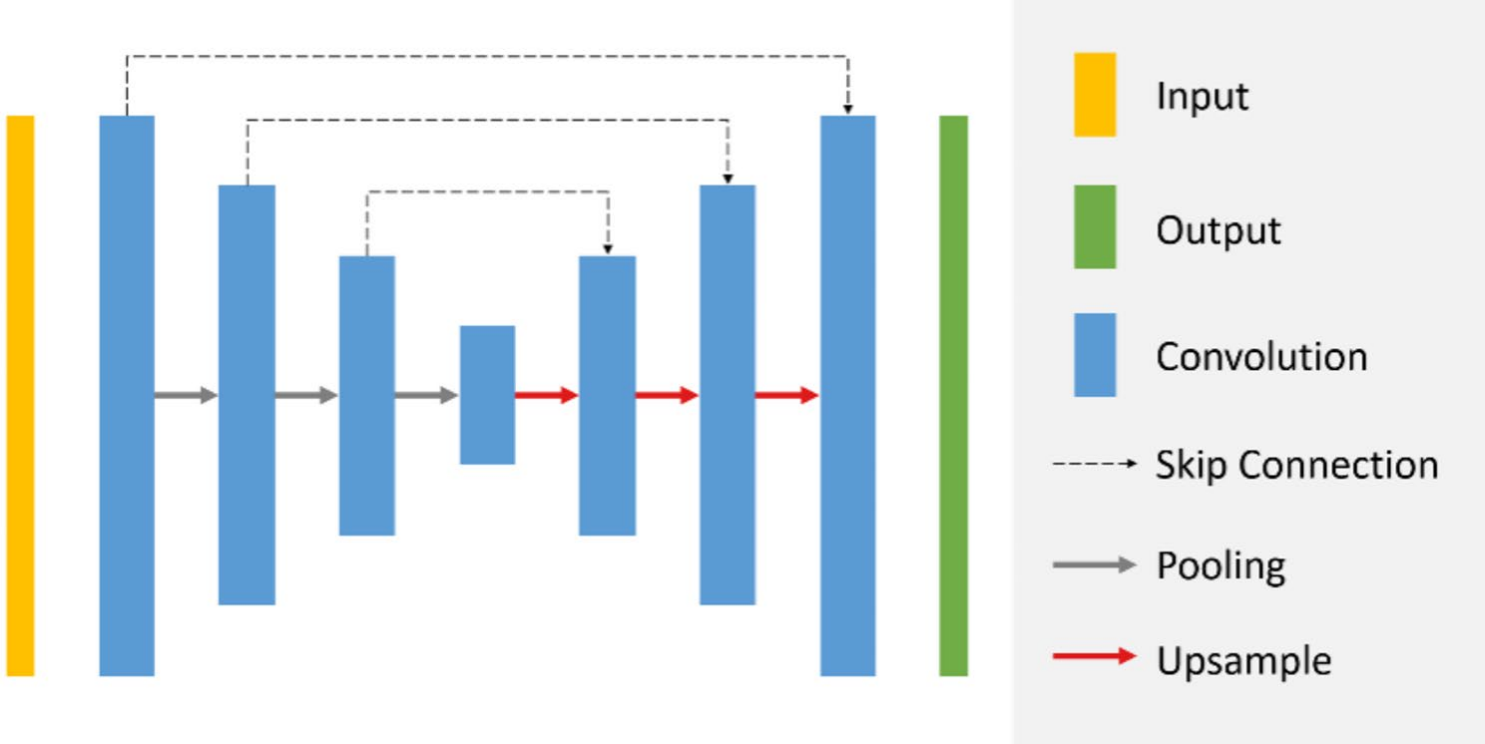
Automatic segmentation network structure

To significantly reduce the interobserver variability inherent in manual segmentation and to increase both the reproducibility and reliability of our study, we developed a fully automatic segmentation method based on the convolutional neural network U-Net [21]. The architecture of the network is illustrated in Fig. 4. Additional details regarding the training process and the evaluation results of the models can be found in the supplementary material.

### Radiomics feature extraction

In this study, all MRI scans were thick-slice scans, which led to significant anisotropy issues. To ensure clear visibility of both muscle and fat distributions, we selected an axial layer of the mid-segment MRI at the thickest part of the gluteus maximus muscle to extract radiomic features, typically at the level of the hip joint or slightly proximal to it. Prior to feature extraction, all the data underwent Z score normalization and were resampled to a uniform resolution of 1 mm² via linear interpolation to ensure consistency. To minimize segmentation errors and prevent the inclusion of nontarget tissues, an erosion process was applied to the segmentation results via a 3 × 3 square structuring element with 1 iteration.

Radiomics features were extracted using PyRadiomics [22] from MRI Dixon T2-weight sequences, including in-phase, opposed-phase, water, fat, and fat fractions. The feature set included 93 attributes across various categories: first-order statistics, Gray level co-occurrence matrix (GLCM), Gray level dependence matrix (GLDM), Gray level run length matrix (GLRLM), Gray level size zone matrix (GLSZM), and Neighboring gray tone difference matrix (NGTDM). A total of 465 features were extracted from these five image types.

### Feature selection

To ensure the reproducibility of features, reduce feature dimensionality, and enhance the robustness of the classification model, we adopted the following steps for feature selection. First, we computed the intragroup correlation coefficients (ICCs) for six regions of interest (ROIs) derived from both manual and automated segmentations, along with their adjacent axial slices. We retained only those features with an ICC value greater than 0.75 to ensure high repeatability. Subsequently, we used the Mann‒Whitney U test to identify features exhibiting significant differences between DMD and BMD, thus eliminating features with minimal contributions to distinguishing the two subtypes. Simultaneously, we filtered out features with a Pearson correlation greater than 0.9 to mitigate collinearity. For each group of features with a Pearson correlation greater than 0.9, we retained only one feature to eliminate redundancy. Finally, the Least Absolute Shrinkage and Selection Operator (LASSO) method was applied to select features that significantly contributed to the classification task.

### Model building and evaluation

In this study, we utilized five supervised ML methods for classification on the basis of the selected features. These methods include random forest (RF), support vector machine (SVM), logistic regression (LR), multilayer perceptron (MLP), and K-nearest neighbors (K-NN). The optimal hyperparameter configurations for each technique were established through a grid search. Following the identification of these optimal settings, we evaluated the efficacy of the models via a 5-fold cross-validation strategy.

Since our study did not include healthy subjects, we defined DMD as the positive class and BMD as the negative class. Accordingly, the following definitions were applied to the classification outcomes: true positive (TP) refers to a DMD case correctly predicted as DMD; true negative (TN) refers to a BMD case correctly predicted as BMD; false positive (FP) is a BMD case incorrectly predicted as DMD; and false negative (FN) is a DMD case incorrectly predicted as BMD.

The evaluation metrics included accuracy, sensitivity, specificity, precision, the F1 score, the receiver operating characteristic (ROC) curve, and the area under the curve (AUC). Definitions for these metrics, along with the related formulas, are provided in the supplementary materials.

### Statistical analysis

All the statistical analyses were conducted via SciPy [23]. Continuous variables were compared via the Mann‒Whitney U test, whereas categorical variables were analyzed via the chi‒square test. A p value of less than 0.05 was considered to indicate statistical significance. The development and implementation of machine learning models were performed via scikit-learn [24].

## Results

### Demographic characteristics

The baseline characteristics and Mercuri scores of the 62 patients included in the study are detailed in Table 1. No significant statistical differences were observed in age between the DMD and BMD groups. Given the genetic nature of the disease, all the study participants were male. Additionally, there were significant statistical differences in the Mercuri scores and edema scores between the two groups.

**Table 1:** Baseline characteristics of the study population, mercuri scores and edema scores (means ± standard deviations; * indicates statistically significant differences)

Characteristics	All Patients(*n* = 62)	DMD(*n* = 41)	BMD(*n* = 21)	*P* value
Age(month)	49.4 ± 6.89	49.5 ± 7.31	49.0 ± 6.15	0.64
Mercuri score				0.007*
0	4	2	2	
1	22	9	13	
2	23	17	6	
3	11	11	0	
4	2	2	0	
Edema score				0.017*
0	35	18	17	
1	24	20	4	
2	3	3	0	
3	0	0	0	
4	0	0	0	

### Reliability of feature extraction

**Fig. 5:**
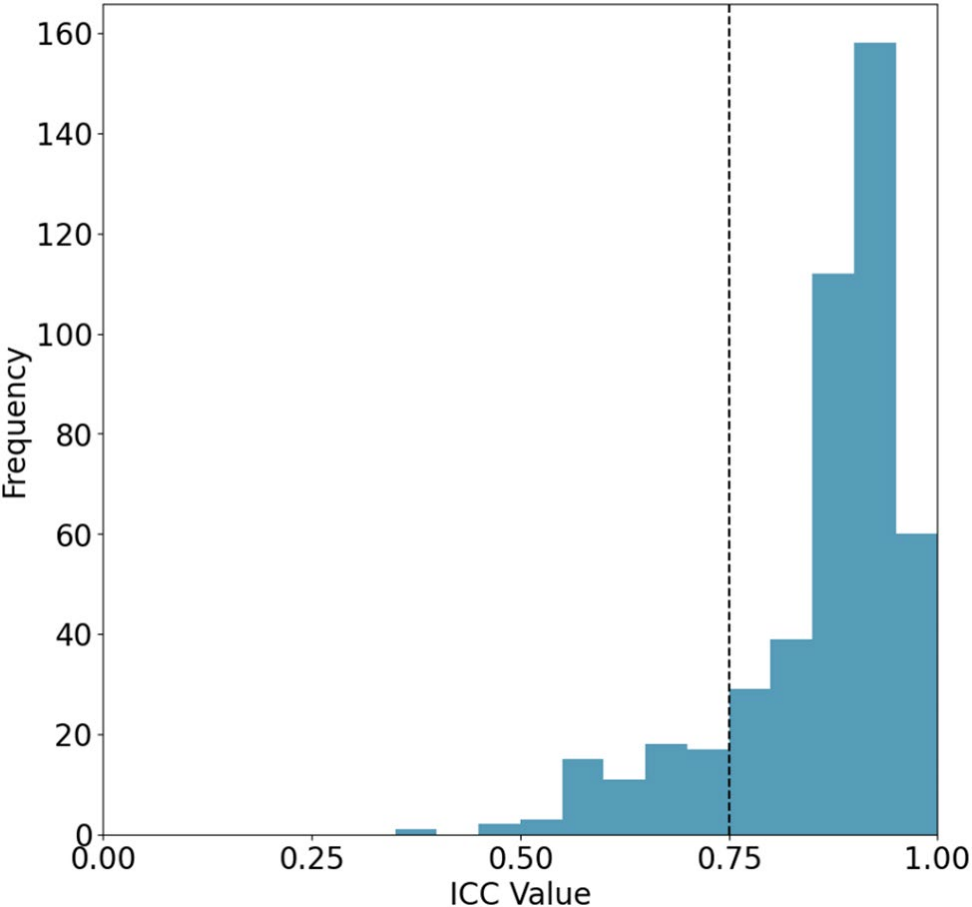
Distribution of feature ICC values. The vertical line represents the threshold at 0.75

To enhance the reliability of radiomics feature extraction and ensure the reproducibility of our study, we selected not only primary gluteus maximus slices but also two adjacent axial slices, resulting in a total of six ROIs. The features extracted from the ROIs were used to calculate the ICC, and the results are displayed in Fig. 5. We set a threshold of an ICC greater than 0.75 for high reliability and retained only those features that met this criterion, for a total of 398 features.

### Feature selection results

**Fig. 6:**
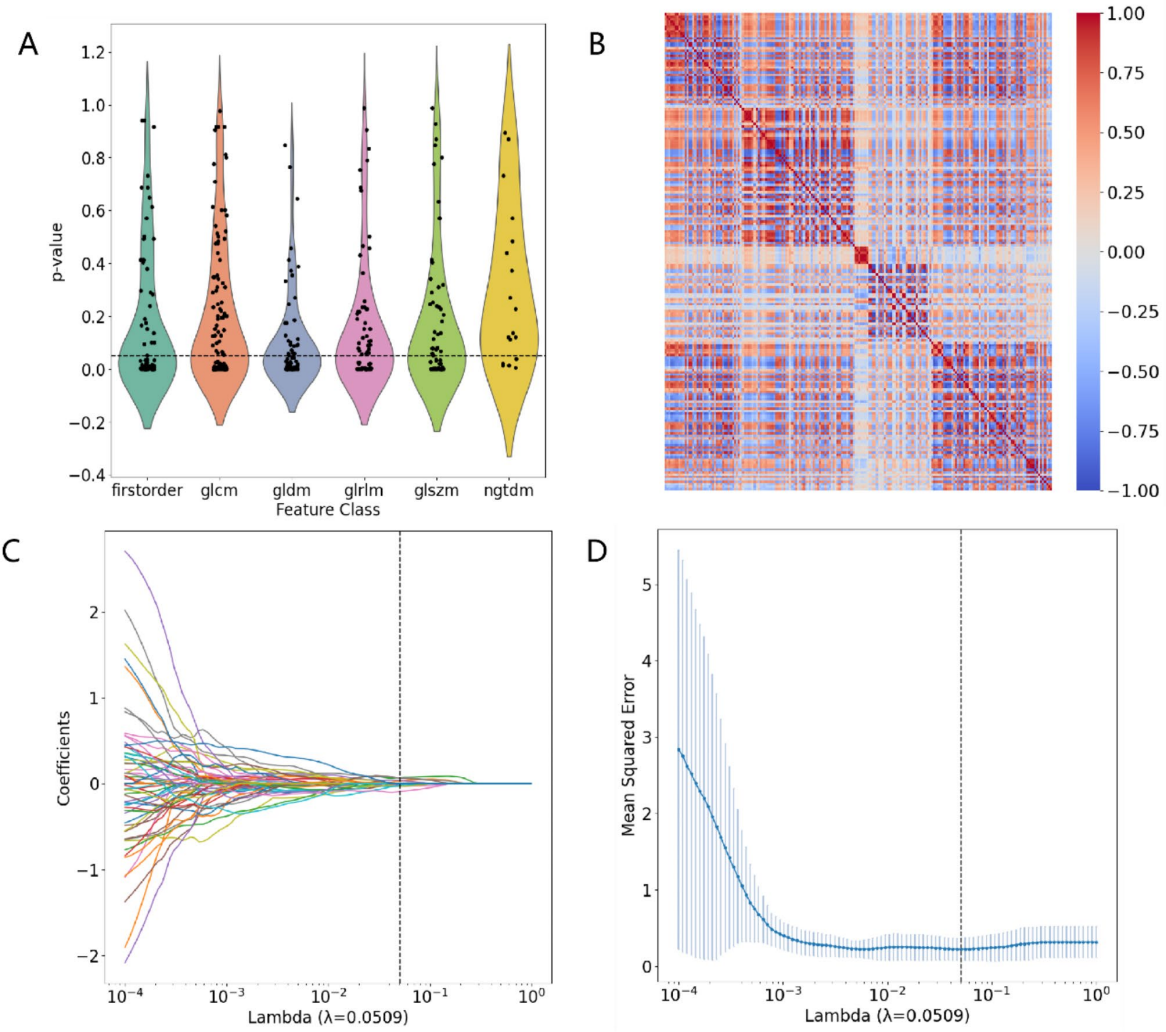
Feature Selection Process. (**A**) Distribution of p values from the Mann‒Whitney U test. (The horizontal line represents the filtering threshold at *p* = 0.05.) (**B**) Pearson correlation heatmap. (**C**) Coefficient path diagram from LASSO regression. (**D**) LASSO 5-fold cross-validation. (The vertical lines in panels C and D indicate the optimal lambda coefficient.)

The process of feature selection is detailed in Fig. 6. First, we used the Mann‒Whitney U test to filter out features that did not show statistically significant differences. Subsequently, we removed features with a collinearity coefficient greater than 0.9 through Pearson correlation analysis. Finally, we applied the LASSO regression method with five-fold cross-validation to select all features with nonzero coefficients for modeling. The final list of selected features is presented in Table 2, and detailed explanations and analyses of these features are included in the supplementary materials.

**Table 2:** Names of selected features

Image	Feature name
in-phase	firstorder_Median
	glcm_InverseVariance
	glszm_LargeAreaEmphasis
opp-phase	gldm_DependenceEntropy
water	firstorder_90Percentile
	glcm_MaximumProbability
	glcm_InverseVariance
	glszm_GrayLevelNonUniformity
	glszm_LargeAreaEmphasis
fat	gldm_LargeDependenceLowGrayLevelEmphasis
fat fraction	glcm_ClusterShade

### Clinical and model judgment performance

The confusion matrix for the radiologist evaluation of BMD and DMD using only MR images is shown in Table 3. Evaluations based on MRI alone still have significant limitations. A substantial number of BMD patients were misclassified as DMD patients, with 17 actual BMD cases being incorrectly predicted as DMD (FP). On the other hand, a small number of DMD patients were misclassified as BMD patients, with only 2 actual DMD cases being incorrectly predicted as BMD (FN).

**Table 3:** Confusion matrix for radiologist evaluation

	Actual DMD	Actual BMD
Predicted DMD	39	17
Predicted BMD	2	4

**Fig. 7:**
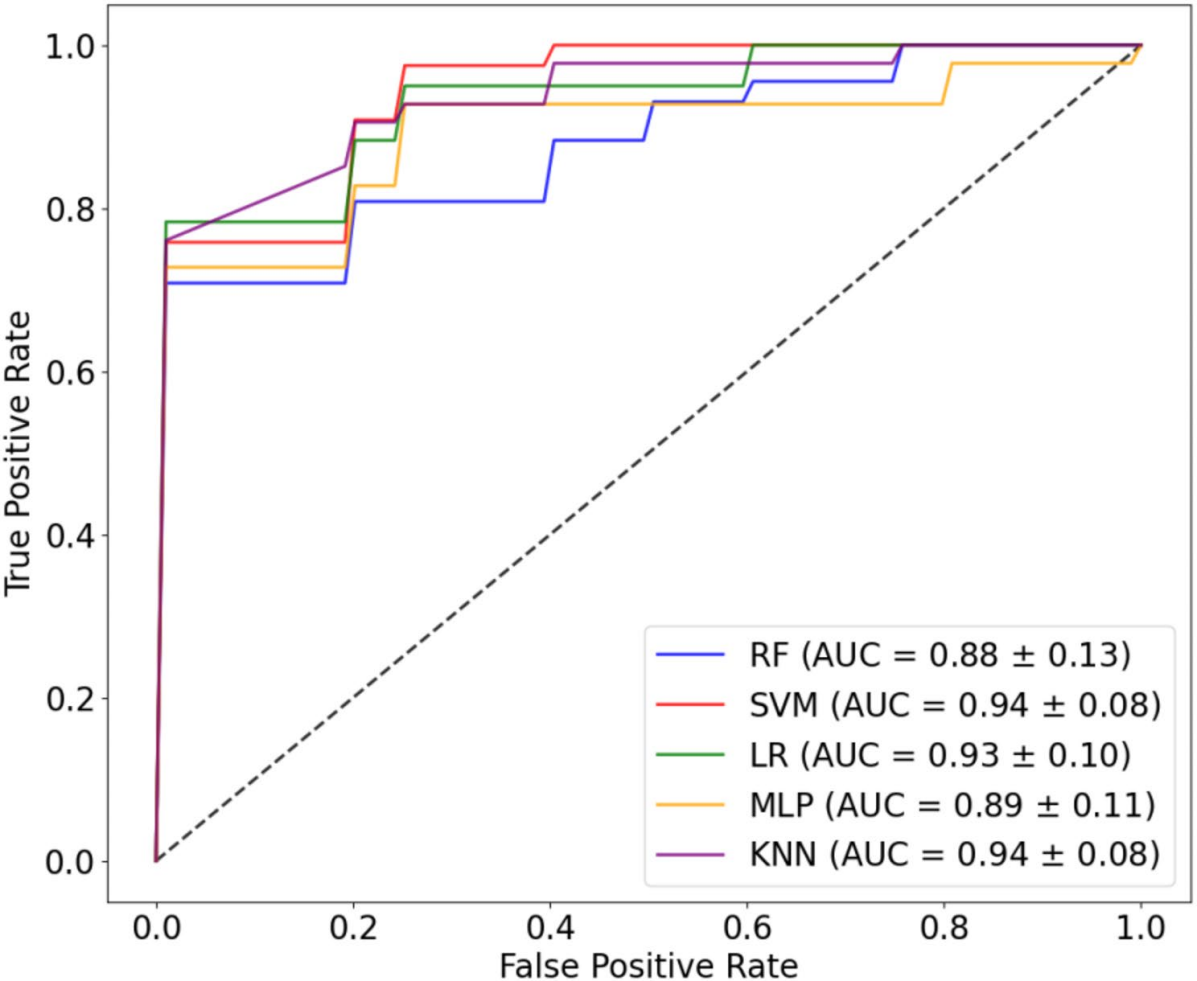
ROC curve (mean ± std)

We utilized five ML algorithms to classify BMD and DMD based on selected features. These algorithms include RF, SVM, LR, MLP and KNN. Each model underwent a grid search optimization process and was evaluated using five-fold cross-validation to assess its performance. The classification performance is presented in Table 4 and Fig. 7.

The radiologists’ sensitivity (95.1%) is notably higher than that of any of the ML models, which is mainly because most patients are classified as DMD, resulting in fewer FNs. However, the specificity of the radiologists is very low (19.0%), as many BMD patients are misclassified as DMD. On the other hand, the ML models demonstrate much higher specificity, meaning that they are better at correctly identifying BMD cases than radiologists are.

In terms of overall performance, the ML models outperform radiologists in terms of accuracy (81.2-90.6% vs. 69.4%), specificity (71.0-86.0% vs. 19.0%), and F1 score (85.2-92.6% vs. 80.5%) while maintaining relatively high sensitivity (85.6-95.0% vs. 95.1%). Compared with the radiologists, the ML models showed significant improvement in specificity, with the MLP achieving 86.0%, indicating a better ability to correctly identify BMD cases without misclassifying them as DMD. In particular, the AUC of SVM and KNN reached 0.94, which is higher than those of the other models. This highlights the ability of ML models to provide a more balanced and accurate diagnostic performance than radiologists do.

**Table 4:** Radiologist performance and ML model performance (mean ± standard deviation)

Methods	Accuracy	Sensitivity	Specificity	F1 score
Radiologists	69.4%	95.1%	19.0%	80.5%
RF	81.2% ± 14.1%	85.6% ± 14.2%	72.0% ± 16.9%	85.2% ± 11.9%
SMV	87.4% ± 10.3%	90.3% ± 14.5%	81.0% ± 9.7%	89.7% ± 9.8%
LR	87.2% ± 9.6%	95.0% ± 10.0%	71.0% ± 24.8%	90.6% ± 7.4%
MLP	90.6% ± 9.0%	92.8% ± 9.9%	86.0% ± 11.6%	92.6% ± 7.5%
KNN	85.9% ± 12.4%	92.8% ± 9.9%	71.0% ± 29.4%	89.8% ± 9.0%

## Discussion

DMD and BMD are rare X-linked recessive dystrophin-associated neuromuscular disorders, occurring in approximately 4.8 per 100,000 live male births [25]. This rarity significantly complicates the collection of substantial research data. Clinical manifestations in the early stages of the disease are often subtle and difficult to detect, leading patients to seek medical attention only after rapid disease progression [26], which complicates data collection efforts.

The treatment and management of DMD and BMD are very different, so it is important to differentiate between DMD and BMD at an early stage. Currently, numerous studies have utilized muscle MRI to analyze muscular dystrophy, including approaches such as employing deep learning techniques to identify patients with muscular dystrophy and using convolutional neural networks to classify dystrophinopathy subtypes [29, 41]. However, most of these studies have focused primarily on differentiating between healthy individuals and patients, and they often do not consider the stage of disease progression [27–30]. In later stages of the disease, when symptoms and effects are more pronounced, it becomes relatively easier to differentiate between the two types of muscular dystrophy. There are also studies showing that serum creatinine (SCRN) can distinguish DMD and BMD in patients aged ≤ 3 years [34]. To our knowledge, our study is the first to employ radiomics and ML techniques to differentiate between DMD and BMD at an early stage based on MRI.

We included 62 patients whose diagnostic details were initially unclear at the time of their first examination. After their conditions progressed to more advanced stages and a definitive diagnosis was established, we conducted a retrospective review of their early MRI sequences. In the clinical evaluations, significant statistical differences were observed in the gluteus maximus edema scores and Mercuri scores between patients diagnosed with BMD and those diagnosed with DMD. Among the 62 patients assessed, 56 were classified as DMD, and 39 were correctly diagnosed out of 42 suspected cases. In contrast, only 6 patients were classified as having BMD, with 4 correct diagnoses out of 21 suspected cases. The radiologist’s evaluation specificity was only 19.0%. Many BMD patients are misdiagnosed with DMD, which may be due to the subjective nature of the diagnosis. One reason for this is that BMD is much less common than DMD in reality. This low specificity of using traditional clinical methods to interpret MRI data does not provide sufficient evidence for the early classification of these two subtypes, which also explains why imaging has historically been of little use in diagnosis [4]. However, this does not imply that MRI alone is incapable of distinguishing between them. Instead, the difficulty in classification most likely stems from the inherent limitations of visual assessment and the subjectivity of radiologists.

Radiomics methods can extract a large number of high-dimensional features from images, allowing for further detailed analysis. Deep learning-based feature extraction methods are also among the current research hotspots and have achieved good performance in medical image classification tasks [30, 44, 45]. However, effective neural network-based feature extractors require a large amount of training data. Based on the reasons mentioned above, we employed radiomics to extract an array of high-throughput information from MR images, which is information that traditional visual assessment fails to detect. Subsequently, we utilized ML methods to develop classification models. Our results improved diagnostic accuracy: the F1 scores of all our classification models ranged from 85.2 to 92.6%. Our model effectively distinguished between the two diseases at an early stage, demonstrating superior performance compared with conventional clinical evaluations.

In radiomics research, segmentation of the ROI is an essential and time-consuming process critical for achieving the study’s objectives. To enhance the reproducibility and operational efficiency of our research, we developed and trained an automated deep learning tool specifically designed for segmentation tasks. This tool effectively reduces the impact of human variability and significantly improves both the efficiency and consistency of the segmentation process. We have made this tool publicly accessible to facilitate further research.

Our research demonstrated that radiomics methods utilizing Dixon sequences can effectively differentiate early-stage DMD and BMD. The accuracy, specificity, and F1 scores of all the classification models were higher than those of the radiologist diagnosis method based on MR images. This finding indicates that MRI, when augmented with advanced radiomic techniques, can provide reliable evidence for distinguishing between DMD and BMD. Although there are currently no effective treatments for these genetic disorders, early diagnosis can offer patients improved management and facilitate potential treatment planning [31].

Although our research successfully developed a method for differentiating early-stage DMD and BMD, it is necessary to recognize the limitations of our study. First, one of the main limitations of this study is its generalizability, which is influenced by the relatively small sample size and the rarity of DMD and BMD cases. Typically, researchers use external datasets to evaluate the generalizability of their models [43]. In our study, we attempted a similar approach by testing the model on data obtained from a different 3.0T MRI scanner (Philips) at the same institution. However, the results indicated that the model’s generalization performance was suboptimal. Upon reviewing the images, we noticed that the Hounsfield unit distribution differed between the MRI scanners, which could be attributed to variations in imaging parameters. Unfortunately, we do not have sufficient data to correct these discrepancies, which affects the model’s ability to generalize effectively. Second, our study did not incorporate clinical presentations, motor function tests, or genetic analyses, all of which are crucial for ensuring accurate diagnoses. The inclusion of these elements in a comprehensive classification model could significantly increase diagnostic precision. Finally, our imaging analysis was confined to the gluteus maximus, even though DMD and BMD can affect various other muscles that may not show noticeable signs on MRI but are critical for comprehensive disease assessment.

In the future, our research will concentrate on establishing multicenter collaborations to collect more data and enhance annotation detail, which is expected to substantially enhance the breadth and precision of our study.

## Conclusion

The results confirm that the radiomics and ML methods based on Dixon sequence imaging can effectively differentiate between DMD and BMD in the early stages, exhibiting a diagnostic performance that surpasses that of traditional clinical assessments. Our study provides an efficient and reliable tool for the early differentiation of these two muscular disorders, potentially enabling more personalized treatment plans for individuals with muscular dystrophy.

## Electronic supplementary material

Below is the link to the electronic supplementary material.

Supplementary Material 1

## Data Availability

We provide the code and more experimental details in the supplementary materials. The raw data are also available from the corresponding author upon reasonable request. The requests to access the data should be sent to li_33@126.com.
