# Supplementary material for: Machine learning-based radiomics using MRI to differentiate early-stage Duchenne and Becker muscular dystrophy in children
Source: BMC Musculoskelet Disord. 2025 Mar 22;26:287. doi: 10.1186/s12891-025-08538-7 (PMC11929326; doi:10.1186/s12891-025-08538-7)
Supplement: Supplementary file 1 — Supplementary Material 1 [file 12891_2025_8538_MOESM1_ESM.pdf]

Part 1 Radiomics research process and code

All our codes are implemented using Python 3.8 and its extension libraries. We provide the idea and code of the whole process. You may need to modify the code to apply your own data. We will be happy to provide you with guidance. You can contact the corresponding author.

1. Preprocessed

Image preprocessing consists of three steps: normalization, resampling, and erosion.

Normalization: Normalize the image by Z-score

```
import SimpleITK as sitk
import numpy as np
# Read the NIfTI image
image_path = 'path_to_your_image.nii'
image = sitk.ReadImage(image_path)
# Convert the SimpleITK image to a NumPy array for easier manipulation
image_array = sitk.GetArrayFromImage(image)
# Calculate the mean and standard deviation of the image
mean = np.mean(image_array)
std_dev = np.std(image_array)
# Apply Z-score normalization
normalized_image_array = (image_array - mean) / std_dev
# Convert the normalized NumPy array back to a SimpleITK image
normalized_image = sitk.GetImageFromArray(normalized_image_array)
# Preserve the original metadata (spacing, origin, direction)
normalized_image.SetSpacing(image.GetSpacing())
normalized_image.SetOrigin(image.GetOrigin())
normalized_image.SetDirection(image.GetDirection())
# Save the normalized image
output_path = 'normalized_image.nii'
sitk.WriteImage(normalized_image, output_path)
print(f"Normalized Image saved to: {output_path}")
```

Resampling: We resample the image pixels to (1mm, 1mm)

```
import SimpleITK as sitk
# Read the NIfTI image
image_path = 'path_to_your_image.nii'
image = sitk.ReadImage(image_path)
# Get the original size and spacing of the image
original_size = image.GetSize()  # Original image size (width, height)
original_spacing = image.GetSpacing()  # Original pixel spacing (in mm)
print(f"Original Size: {original_size}")
print(f"Original Spacing: {original_spacing}")
# Define the target spacing (1mm x 1mm)
```

```
new_spacing = (1.0, 1.0)
# Calculate the new image size based on the target spacing
new_size = [
    int(round(original_size[0] * original_spacing[0] /
new_spacing[0])),  # Calculate new width
    int(round(original_size[1] * original_spacing[1] /
new_spacing[1]))   # Calculate new height
]
# Resample the image to the new spacing and size
resampled_image = sitk.Resample(
    image,
    new_size,              # New image size
    sitk.Transform(),      # No transformation
    sitk.sitkLinear,       # Use linear interpolation
    image.GetOrigin(),     # Keep the original origin
    new_spacing,           # Set the new spacing
    image.GetDirection(),  # Keep the original orientation
    0,                     # Default value for outside pixels
(background)
)
# Save the resampled image to a new file
output_path = 'resampled_image.nii'
sitk.WriteImage(resampled_image, output_path)
print(f"Resampled Image saved to: {output_path}")
```

Erosion: We use a 3x3 square structure to erosion the label with 1 iterations.

```
import SimpleITK as sitk
import numpy as np
from scipy import ndimage
label = sitk.ReadImage('path/to/your/label.nii')  # Replace with your
NIfTI file path
label_array = sitk.GetArrayFromImage(label)  # Converts the image to a
3D NumPy array (Z, Y, X)
label_array = np.where(label_array > 0, 1, 0)  # Any non-zero value is
set to 1 (foreground), else 0 (background)
label_array_erode = ndimage.binary_erosion(label_array,
iterations=1).astype(np.uint8)
label_erode = sitk.GetImageFromArray(label_array_erode)  # Converts the
array back to SimpleITK image format
label_erode.SetSpacing(label.GetSpacing())  # Ensure the spatial
resolution (spacing) is preserved
label_erode.SetOrigin(label.GetOrigin())    # Ensure the origin
(position in space) is preserved
sitk.WriteImage(label_erode, 'path/to/save/eroded_label.nii')  # Specify
the output file path
```

2. Radiomics Feature Extract

We used the Radiomics library in Python to extract radiomics features. We used all features and set the binWidth of in-phase, inverse, water, and fat to 0.1 and the binWidth of fat fraction to 0.01, and its parameters and code were set as follows

```
def get_feature(image_path,mask_path,extractor):
    # print(image_path,mask_path)
    image=sitk.ReadImage(image_path)
    mask=sitk.ReadImage(mask_path)
    feature=extractor.execute(image,mask)
    for key in list(feature.keys()):
        if 'diagnostics' in key:
            del feature[key]
        if 'shape2D' in key:
            del feature[key]
    feature=pd.DataFrame.from_dict(feature,orient='index').T
    if '0000' in image_path:
        name='in'
    elif '0001' in image_path:
        name='op'
    elif '0002' in image_path:
        name='w'
    elif '0003' in image_path:
        name='f'
    elif '0004' in image_path:
        name='ff'
    for i in feature.columns:
        feature.rename(columns={i:name+'_'+i},inplace=True)
    return feature
from radiomics import featureextractor
extractor = featureextractor.RadiomicsFeatureExtractor()
extractor.enableAllFeatures()
extractor.settings['binWidth'] = 0.1
in_feature=get_feature(image_in_path[j],label_roi[j],extractor)
op_feature=get_feature(image_op_path[j],label_roi[j],extractor)
w_feature=get_feature(image_w_path[j],label_roi[j],extractor)
f_feature=get_feature(image_f_path[j],label_roi[j],extractor)
extractor.settings['binWidth'] = 0.01
ff_feature=get_feature(image_ff_path[j],label_roi[j],extractor)
```

3. Intraclass Correlation Coefficient:

We calculated the ICC of the six ROIs and retained the features with an ICC greater than 0.75.

```
import pingouin as pg
import pandas as pd
```

```
# Example function to calculate ICC for all features
def calculate_icc_and_filter(features_df):
    icc_values = []
    # Iterate through each feature/column in the DataFrame
    for feature in features_df.columns:
        # Get the ICC for the current feature (across the different
conditions)
        icc_result = pg.intraclass_corr(data=features_df,
targets='sample_id', raters='condition', ratings=feature)
        # Extract ICC value (ICC is usually located in the "ICC" column
of the result)
        icc = icc_result['ICC'][0]  # We assume there's only one row for
each feature
        icc_values.append((feature, icc))
    # Convert list of ICC values to DataFrame
    icc_df = pd.DataFrame(icc_values, columns=['Feature', 'ICC'])
    # Filter features with ICC > 0.75
    filtered_features = icc_df[icc_df['ICC'] > 0.75]
    return filtered_features
# Example DataFrame for demonstration (replace with actual feature
data)
# Convert to pandas DataFrame
features_df = pd.DataFrame(feature)
# Set 'sample_id' as the index for easier manipulation
features_df.set_index('sample_id', inplace=True)
# Now we call the function to calculate ICC and filter features with
ICC > 0.75
filtered_features = calculate_icc_and_filter(features_df)
# Print the filtered features with ICC > 0.75
print(filtered_features)
```

4. U-test.

Filter out features that have no statistically significant difference

```
import pandas as pd
import seaborn as sns
import matplotlib.pyplot as plt
import numpy as np
# Extract p-values for each feature class from the feature_p DataFrame
firstorder =
feature_p[feature_p['Feature'].str.contains('firstorder')]['p_value']
glcm = feature_p[feature_p['Feature'].str.contains('glcm')]['p_value']
gldm = feature_p[feature_p['Feature'].str.contains('gldm')]['p_value']
glrlm =
feature_p[feature_p['Feature'].str.contains('glrlm')]['p_value']
```

```
glszm =
feature_p[feature_p['Feature'].str.contains('glszm')]['p_value']
ngtdm =
feature_p[feature_p['Feature'].str.contains('ngtdm')]['p_value']
# Create a dictionary of the p-values
data = {
    'firstorder': firstorder.values,
    'glcm': glcm.values,
    'gldm': gldm.values,
    'glrlm': glrlm.values,
    'glszm': glszm.values,
    'ngtdm': ngtdm.values
}
# Create a DataFrame from the dictionary
df = pd.DataFrame({key: pd.Series(value) for key, value in
data.items()})
# Convert the DataFrame to long format
df_melt = df.melt(var_name='Feature Class', value_name='p-value')
# Set the figure size
plt.rcParams.update({'font.size': 20})
plt.figure(figsize=(10, 10))
# Draw a horizontal line at y = 0.05
plt.axhline(y=0.05, color='black', linestyle='--')
# Create the violin plot
sns.violinplot(x='Feature Class', y='p-value', data=df_melt,
inner=None, palette='Set2')
# Overlay the strip plot to show individual data points
sns.stripplot(x='Feature Class', y='p-value', data=df_melt,
jitter=True, color='k', alpha=1)
# Remove grid lines
plt.grid(False)
# Display the plot
plt.show()
```

5. Pearson:

Remove features with high collinearity

```
    import pandas as pd
import numpy as np
import seaborn as sns
import matplotlib.pyplot as plt
# Load the features data
feature_df = pd.read_csv('dataset/feature/features.csv')
file_name = feature_df['file_name']
label = feature_df['label']
# Calculate the Pearson correlation coefficients between features
```

```python
corr = feature_df.corr()
# Plot the correlation heatmap
plt.figure(figsize=(10, 10))
plt.gca().xaxis.set_visible(False)
plt.gca().yaxis.set_visible(False)
sns.heatmap(corr, annot=False, cmap='coolwarm', fmt=".2f", cbar=True,
vmin=-1, vmax=1)
plt.show()
# Filter out features with correlation coefficients greater than 0.9
corr_matrix = feature_df.corr().abs()
# Extract the upper triangular matrix of correlations (to avoid
duplicate calculations)
upper = corr_matrix.where(np.triu(np.ones(corr_matrix.shape),
k=1).astype(bool))
# Set the correlation threshold for filtering
threshold = 0.9
# Find the high correlation feature pairs
high_correlation_pairs = [(column, row) for column in upper.columns for
row in upper.index
                          if abs(upper[column][row]) > threshold]
# Set to track features to drop
to_drop = set()
# Remove one feature from each pair with high correlation, keeping the
one with higher variance
for col, row in high_correlation_pairs:
    if feature_df[col].var() > feature_df[row].var():
        to_drop.add(row)
    else:
        to_drop.add(col)
# Create a filtered feature DataFrame by dropping the highly correlated
features
data_filtered = feature_df.drop(columns=to_drop)
# Recompute the correlation matrix for the filtered features
filtered_correlation_matrix = data_filtered.corr(method='pearson')
# Ensure no correlation is above the threshold in the filtered data
assert not any(abs(filtered_correlation_matrix[col][row]) > threshold
              for col in filtered_correlation_matrix.columns
              for row in filtered_correlation_matrix.index
              if col != row)
# Print the shape of the filtered correlation matrix
print(f"Filtered correlation matrix shape:
{filtered_correlation_matrix.shape}")
# Plot the heatmap of the filtered correlation matrix
sns.heatmap(filtered_correlation_matrix, annot=False, cmap='coolwarm')
```

```
# Print the remaining features after filtering
print("Filtered feature set:", data_filtered.columns)
# Save the filtered feature names to a CSV file
filtered_features = pd.DataFrame(data_filtered.columns,
columns=['Feature'])
filtered_features.to_csv('dataset/feature/Pearson.csv', index=False)
```

6. Lasso:

With grid search of the regularization parameter (alpha) using 5-fold cross-validation and negative mean squared error as the scoring metric, with a maximum of 10,000 iterations for convergence.

```
import matplotlib.pyplot as plt
from sklearn.linear_model import Lasso
from sklearn.model_selection import GridSearchCV
from sklearn.preprocessing import StandardScaler
import numpy as np
import pandas as pd
# Define the feature set (X) and target label (y)
X = feature_df
y = label
# Standardize the feature set to have zero mean and unit variance
scaler = StandardScaler()
X_scaled = scaler.fit_transform(X)
# Define a range of alpha (λ) values to explore for regularization
alphas = np.logspace(-4, 0, 100)
# Initialize the Lasso model
lasso = Lasso(max_iter=10000)
# Use GridSearchCV for cross-validation to find the best alpha (λ) that
minimizes MSE
param_grid = {'alpha': alphas}
lasso_cv = GridSearchCV(lasso, param_grid, cv=5,
scoring='neg_mean_squared_error')
lasso_cv.fit(X_scaled, y)
# Print the best alpha (λ) value found by cross-validation
best_alpha = lasso_cv.best_params_['alpha']
print("Best alpha:", best_alpha)
# Get the Mean Squared Error (MSE) and standard deviation of MSE for
each fold
mse = -lasso_cv.cv_results_['mean_test_score']
std = lasso_cv.cv_results_['std_test_score']
# Plot the cross-validation error (MSE) for each alpha (λ) value
plt.figure(figsize=(10,10))
plt.errorbar(alphas, mse, yerr=std, fmt='-o', ecolor='lightsteelblue',
markersize=3, capsize=1, label='Mean Squared Error with Std Dev')
```

```
plt.axvline(lasso_cv.best_params_['alpha'], color='black', linestyle='-
-', label='Best Alpha')
plt.xscale('log')  # Log scale for alpha (λ)
plt.xlabel('Lambda (λ={:.4f})'.format(lasso_cv.best_params_['alpha']))
plt.ylabel('Mean Squared Error')
plt.show()
# Initialize a list to store coefficients for each alpha (λ)
coefficients = []
# For each alpha, train a Lasso model and record the coefficients
for alpha in alphas:
    lasso.set_params(alpha=alpha)
    lasso.fit(X_scaled, y)
    coefficients.append(lasso.coef_)
# Convert the coefficients to a numpy array for easier plotting
coefficients = np.array(coefficients)
# Plot the Lasso path showing how coefficients change with different
alpha values
plt.figure(figsize=(10, 10))
for i in range(coefficients.shape[1]):
    plt.plot(alphas, coefficients[:, i], label=X.columns[i])
plt.axvline(lasso_cv.best_params_['alpha'], color='black', linestyle='-
-', label='Best Alpha')
plt.xscale('log')  # Log scale for alpha (λ)
plt.xlabel('Lambda (λ={:.4f})'.format(lasso_cv.best_params_['alpha']))
plt.ylabel('Coefficients')
plt.show()
# Train the final Lasso model with the best alpha (λ) found
lasso_best = Lasso(alpha=best_alpha, max_iter=10000)
lasso_best.fit(X_scaled, y)
# Get the coefficients from the model with the best alpha (λ)
best_coefficients = lasso_best.coef_
# Select features that have non-zero coefficients (important features)
important_features = X.columns[best_coefficients != 0]
print("Important features:", important_features)
# Plot the important features with their corresponding coefficients
plt.figure(figsize=(10, 10))
important_features_coef = best_coefficients[best_coefficients != 0]
important_features_coef, important_features =
zip(*sorted(zip(important_features_coef, important_features)))
plt.barh(important_features, important_features_coef)
plt.xlabel('Coefficients')
plt.title('Important Features')
plt.show()
# Convert the important features to a list for further use
```

```
important_features = list(important_features)
# Save the important features and their coefficients to a CSV file
lasso_df = pd.DataFrame()
lasso_df['Feature'] = important_features
lasso_df['Coefficient'] = important_features_coef
lasso_df.to_csv('dataset/feature/Lasso.csv', index=False)
```

7. Classification model:

We evaluated the classification performance of five different models using a grid search.

```
from sklearn.ensemble import RandomForestClassifier
param_grid = {
    'n_estimators': [i for i in range(20, 30)],
    'max_depth': [i for i in range(1, 5)]
}
rf = RandomForestClassifier()
from sklearn.metrics import make_scorer, accuracy_score,
precision_score, recall_score, f1_score
scoring = {
    'accuracy': make_scorer(accuracy_score),
    'precision': make_scorer(precision_score),
    'recall': make_scorer(recall_score),
    'f1': make_scorer(f1_score)
}
from sklearn.svm import SVC
rf_cv = GridSearchCV(rf, param_grid, cv=5, scoring=scoring, refit='f1')
rf_cv.fit(X_select_scaled, y)from sklearn.svm import SVC
param_grid = {
    'C': np.logspace(-3, 3, 7),
    'kernel': ['linear', 'poly', 'rbf', 'sigmoid']
}
svm = SVC()
svm_cv = GridSearchCV(svm, param_grid, cv=5, scoring=scoring,
refit='f1')
svm_cv.fit(X_select_scaled, y)
from sklearn.linear_model import LogisticRegression
param_grid = {
    'C': np.logspace(-3, 3, 7),
    'penalty': ['l1', 'l2']
}
lr = LogisticRegression()
lr_cv = GridSearchCV(lr, param_grid, cv=5, scoring=scoring, refit='f1')
lr_cv.fit(X_select_scaled, y)
from sklearn.neural_network import MLPClassifier
param_grid = {
```

```
    'hidden_layer_sizes': [(i,) for i in range(1, 10)],
    'activation': ['identity', 'logistic', 'tanh', 'relu']
}
mlp = MLPClassifier(max_iter=100)
mlp_cv = GridSearchCV(mlp, param_grid, cv=5, scoring=scoring,
refit='f1')
mlp_cv.fit(X_select_scaled, y)
from sklearn.neighbors import KNeighborsClassifier
param_grid = {
    'n_neighbors': [i for i in range(1, 20)],
    'weights': ['uniform', 'distance']
}
knn = KNeighborsClassifier()
# 使用 GridSearchCV 进行交叉验证
knn_cv = GridSearchCV(knn, param_grid, cv=5, scoring=scoring,
refit='f1')
knn_cv.fit(X_select_scaled, y)
```

Finally, we use the best parameters obtained by grid search to perform a five-fold cross validation and report it as the final result.

```
from sklearn.model_selection import cross_val_predict, StratifiedKFold
rf_best = RandomForestClassifier(**rf_cv.best_params_)
svm_best = SVC(**svm_cv.best_params_,probability=True)
lr_best = LogisticRegression(**lr_cv.best_params_)
mlp_best = MLPClassifier(**mlp_cv.best_params_)
knn_best = KNeighborsClassifier(**knn_cv.best_params_)
rf_tprs,rf_aucs,rf_accuracies,rf_recalls,rf_precisions,rf_f1_scores,rf_
ys,rf_y_pred_probs,rf_specificity=cv5(X_select_scaled,y,rf_best)
svm_tprs,svm_aucs,svm_accuracies,svm_recalls,svm_precisions,svm_f1_scor
es,svm_ys,svm_y_pred_probs,svm_specificity=cv5(X_select_scaled,y,svm_be
st)
lr_tprs,lr_aucs,lr_accuracies,lr_recalls,lr_precisions,lr_f1_scores,lr_
ys,lr_y_pred_probs,lr_specificity=cv5(X_select_scaled,y,lr_best)
mlp_tprs,mlp_aucs,mlp_accuracies,mlp_recalls,mlp_precisions,mlp_f1_scor
es,mlp_ys,mlp_y_pred_probs,mlp_specificity=cv5(X_select_scaled,y,mlp_be
st)
knn_tprs,knn_aucs,knn_accuracies,knn_recalls,knn_precisions,knn_f1_scor
es,knn_ys,knn_y_pred_probs,knn_specificity=cv5(X_select_scaled,y,knn_be
st)
```

The above is the entire process of our experiment. You can follow these steps to complete your code to reproduce our method.

Part 2 Discussion on feature extraction methods

In radiomics research, the choice of feature screening method directly affects the performance of the final model. In the author's experience, no single method or combination of screening methods performs best on all models. The selection of feature screening methods should be based on comprehensive considerations of data, tasks, goals, and experimental performance.

First, we explain why we apply other feature selection methods before Lasso in our experiments:

1. Reduce the amount of calculation

In radiomics, the data often contains hundreds or even thousands of features, many of which may be redundant, noisy, or weakly related to the target variable. If Lasso regression is applied directly to such high-dimensional data, not only will the computational complexity be high, but it could also lead to overfitting, as Lasso's regularization might struggle to effectively select truly useful features in a complex feature space. By first using other feature selection methods, redundant features can be removed and the feature dimension can be reduced, allowing Lasso regression to run on a more simplified feature set, thereby improving computational efficiency.

2. Improving the effectiveness of Lasso regression

Lasso regression itself has strong feature selection capability (by shrinking coefficients through L1 regularization), but its performance may not meet expectations when dealing with very high-dimensional feature spaces, especially when features are highly correlated. By using other feature selection methods, you can preemptively remove features that are weakly related to the target variable or redundant. This allows Lasso to select from a more "streamlined" feature set, avoiding unstable selection results in a highly redundant feature space.

3. Avoid multicollinearity between features

In radiomics data, some features may be highly correlated (for example, multiple features derived from different texture calculations may be very similar). If these highly correlated features are directly input into Lasso regression, it could lead to multicollinearity, making the feature selection process unstable. By first using correlation analysis or variance screening, you can remove highly correlated features, thus reducing the impact of multicollinearity. This allows Lasso regression to more effectively select features with stronger independence.

Then we will explain why we used ICC (Intraclass Correlation Coefficient), Pearson correlation, and the U-test for the following reasons:

1. ICC: Improve the reproducibility of features

Since our ROI (Region of Interest) was manually selected from 3D medical images, we used ICC to assess and improve the reproducibility of the features extracted from these images. In radiomics, it is crucial to ensure that the features we are analyzing are consistent and stable, whether they are measured by different raters or across multiple scans. High ICC values indicate that the extracted features are reliable and reproducible, which is essential for ensuring that our results are not influenced by subjective variability.

2. U-test.:

The U-test (Mann-Whitney U test) was used to identify statistically significant differences between two independent groups based on the radiomic features. Since many radiomic features often do not follow a normal distribution, the U-test is an appropriate non-parametric method that allows us to assess whether two groups differ significantly in terms of feature distributions. This helps to ensure that the features we select are informative and capable of distinguishing between different groups, which is crucial for building predictive models and understanding the underlying patterns in the data. This can also effectively improve the efficiency of Lasso.

3. Pearson:

We used Pearson correlation to measure the linear relationships between pairs of features. Radiomic features can often be highly correlated due to the nature of the image data. By calculating the Pearson correlation, we could identify and manage redundant features that convey similar information. This step is essential to reduce multicollinearity in the dataset, which can destabilize models and lead to overfitting. Removing or consolidating highly correlated features allows us to streamline the feature set, making Lasso more stable and efficient.

Finally, the author provides readers with some radiomics feature screening ideas for reference:

1. Consider the characteristics of your data

When selecting features, always take into account the nature and characteristics of your data. For example, in this study, since we introduced a subjective process of selecting ROIs, we used ICC to eliminate this variability. If you have too many features, you can use more or stricter feature screening methods before the final Lasso or similar method to improve the stability of Lasso. The choice of feature extraction methods and screening techniques should align with the data's characteristics to ensure that the most relevant and informative features are selected for your specific problem.

2. If you are not sure whether a method is effective, try them one by one from simple to complex.

Radiomics is not a technology that can be fully explained, and feature screening is inherently a complex process. The impact of each method on the final results can be difficult to predict in advance because it depends on various factors. Given this uncertainty, it's important to conduct preliminary experiments before deciding on a specific method.

3.Try using deep learning methods to extract features

In addition to traditional radiomics feature extraction methods, consider experimenting with deep learning models to extract features directly from the raw image data. Deep learning can potentially uncover complex, non-linear patterns in the data that might not be captured through manual or traditional feature extraction methods. This approach may help improve prediction accuracy and uncover new insights. But it should be noted that training an efficient deep learning model requires a large amount of data. Please consider the amount of your data first.

Part 3 Detailed formula of evaluation metrics

Due to the limited length of the main text, we give here the evaluation index formula used in the manuscript.

Since our study did not include healthy subjects, we defined DMD as the positive class and BMD as the negative class. Accordingly, the following definitions were applied to the classification outcomes: True Positive (TP) refers to a DMD case correctly predicted as DMD; True Negative (TN) refers to a BMD case correctly predicted as BMD; False Positive (FP) is a BMD case incorrectly predicted as DMD; False Negative (FN) is a DMD case incorrectly predicted as BMD.

The evaluation metrics included accuracy, sensitivity, specificity, precision, F1-Score, the Receiver Operating Characteristic (ROC) curve, and Area Under the Curve (AUC). The calculation method is as follows:

$$\text{Accuracy} = \frac{TP + TN}{TP + TN + FP + FN}$$

$$\text{Sensitivity(Recall)} = \frac{\text{TP}}{\text{TP} + \text{FN}}$$

$$\text{Specificity} = \frac{TN}{TN + FP}$$

$$\text{Precision} = \frac{TP}{TP + FP}$$

$$\text{F1-Score} = 2 \times \frac{\text{Precision} \times \text{Sensitivity}}{\text{Precision} + \text{Sensitivity}}$$

The ROC curve is a plot of the true positive rate (sensitivity) versus the false positive rate 1−Specificity

$$\text{False Positive Rate (FPR)} = \frac{FP}{FP + TN}$$

The AUC is the area under the ROC curve and quantifies the overall performance of the model. It is computed by numerical integration of the ROC curve, but it's often represented as:

$$\text{AUC} = \int_0^1 \text{Sensitivity } d(\text{FPR})$$

Part 4 Representative MRI displays

Based on the experience of radiologists and clinicians in our institution, DMD and BMD patients under the age of five years (60 months) are almost indistinguishable based on MRI alone. This is because the disease progresses slowly in the early stages and the degree of fat muscle replacement and edema in both are the same. There may be special cases depending on individual differences. For intuitive display, we have selected representative MRI images for display.

Figure 4.1 is the MRI Dixon sequence of DMD patients and BMD patients, from left to right, they are in phase, reverse, water, and fat. The upper column is a 36-month-old DMD patient. The radiologist scored the Mercuri score of his gluteus maximus as 1 and the edema score as 0, and he was evaluated as DMD. The lower column is a 37-month-old BMD patient. The radiologist scored the Mercuri score of his gluteus maximus as 1 and the edema score as 1, and he was evaluated as DMD.

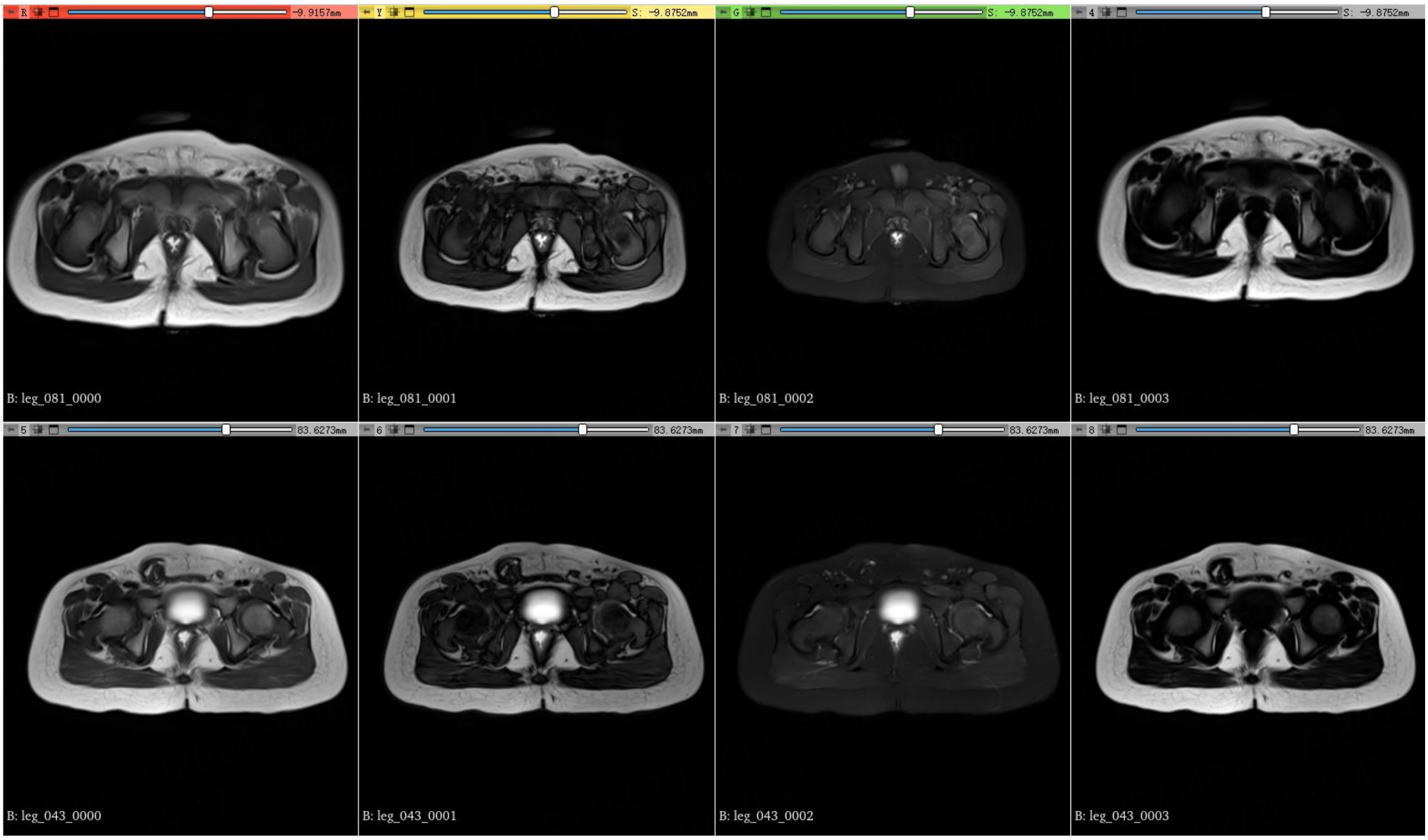

Figure 4.1 The top row shows the MRI of a 36-month-old DMD patient, and the bottom row shows the MRI of a 37-month-old BMD patient.

According to our evaluation results, before 60 months, radiologists would only evaluate patients with BMD if their Mercuri and edema scores were both 0. Therefore,

17 of the 21 patients with BMD were incorrectly diagnosed with DMD in the radiologists' assessment. This means that it is almost impossible to distinguish the two subtypes using MRI alone.

Next, let's look at a group of older examples. Figure 4.2 is the MRI Dixon sequence of DMD patients and BMD patients, from left to right, they are in phase, reverse, water, and fat. The upper column is a 58-month-old DMD patient. The radiologist scored the Mercuri score of his gluteus maximus as 1 and the edema score as 0, and he was evaluated as DMD. The lower column is a 51-month-old BMD patient. The radiologist scored the Mercuri score of his gluteus maximus as 2 and the edema score as 1, and he was evaluated as DMD.

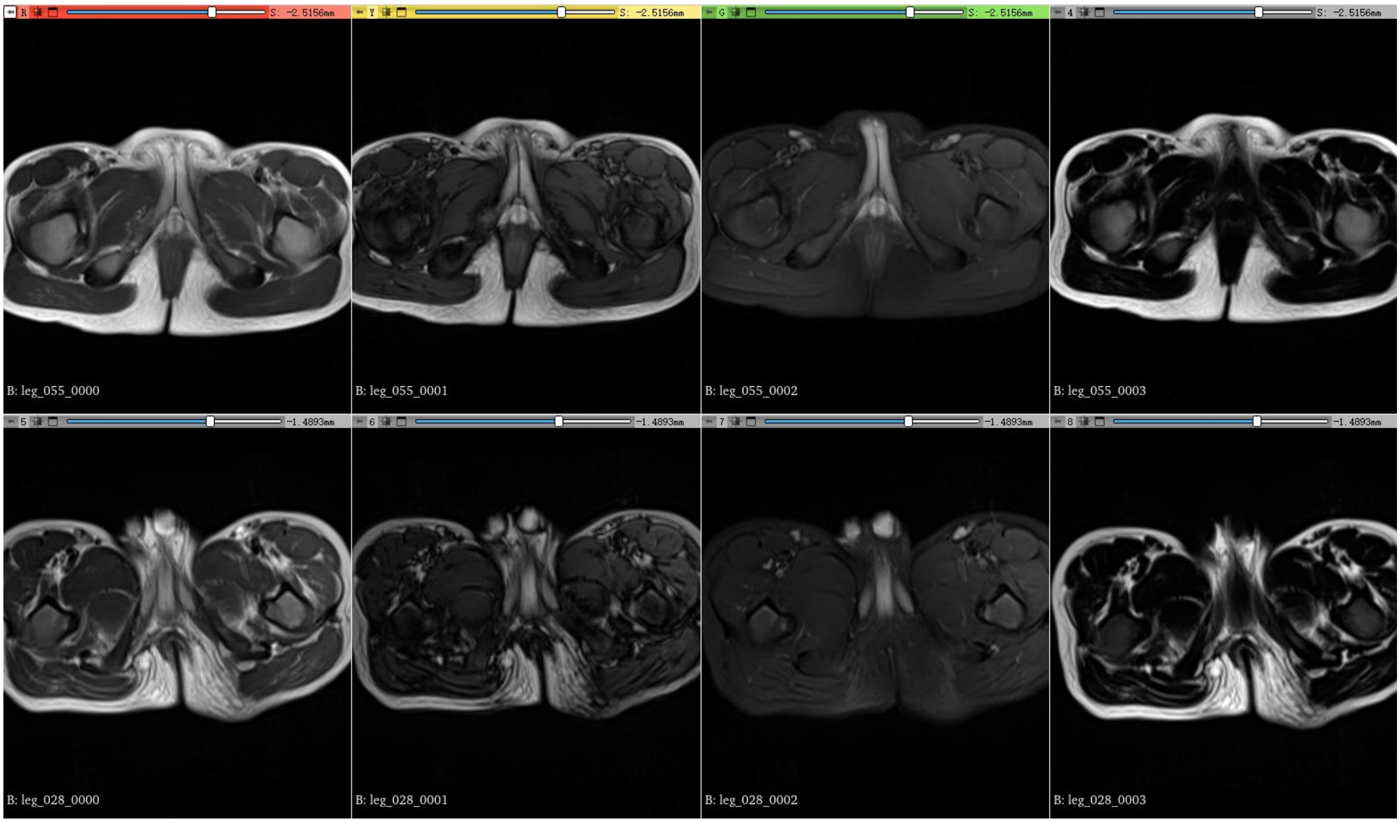

Figure 4.2 The top row shows the MRI of a 58-month-old DMD patient, and the bottom row shows the MRI of a 51-month-old BMD patient.

From our case, we can see that the individual differences between DMD and BMD patients before 60 months old are very obvious, and one of the reasons why radiologists evaluate most patients as DMD is that BMD itself is relatively rare. This also proves the potential value of our radiomics method

Part 5 Training of automatic segmentation network

Our automatic segmentation network is based on nnUnet, and you can find its detailed usage at https://github.com/MIC-DKFZ/nnUNet . We only show here how we use it for automatic segmentation.

1. Training data

The original data is a T2-Weighted Dixon sequence obtained by Siemens scanning. Each patient contains four MRI images (in phase, reverse, water, fat) and manually drawn gluteus maximus annotations. The name and format of the file are shown in Figure 5.1 below.

```
dataset/raw
├──dataset.json
├──image # MRI
│    ├leg_001_0000.nii.gz # IP
│    ├leg_001_0001.nii.gz # OP
│    ├leg_001_0002.nii.gz # W
│    ├leg_001_0003.nii.gz # F
│    ├leg_002_0000.nii.gz
│    ├leg_002_0001.nii.gz
│    ├leg_002_0002.nii.gz
│    ├leg_002_0003.nii.gz
│    ├...
│──manual # ROI
     ├leg_001.nii.gz # label
     ├....
```

Figure 5.1 File format

2. Image preprocessing

When the data is ready, use the following command to preprocess it:

```
nnUNetv2_plan_and_preprocess -d DATASET_ID --verify_dataset_integrity
```

Where DATASET_ID is the dataset id (duh). We recommend --verify_dataset_integrity whenever it's the first time you run this command. This will check for some of the most common error sources!

3. Start training

After successful preprocessing, you can start training U-net on your data using the following command:

```
nnUNetv2_train DATASET_NAME_OR_ID UNET_CONFIGURATION FOLD [additional options, see -h]
```

This is a general command that you may need to adjust based on your dataset. According to the characteristics of our data, we use 2D U-net for training. You can also try 3D.

4. Network prediction

When you have trained your network, you can run the following code to test it on your test set:

```
nnUNetv2_predict -i INPUT_FOLDER -o OUTPUT_FOLDER -d DATASET_NAME_OR_ID -c CONFIGURATION --save_probabilities
```

Because the default training of nnUnet is 5-fold cross validation, you can test it on all the data.

5. Segmentation Accuracy Assessment

nnUnet uses DSC (Dice Similarity Coefficient) to evaluate the accuracy of segmentation result,

$$DSC = \frac{2 \times |A \cap B|}{|A| + |B|}$$

where A and B represent two sample sets, $|A \cap B|$ represents the size of the intersection of set A and set B , $|A|$ and $|B|$ represent the size of set A and set B.

According to the prediction results of nnUnet, the average DSC of our five-fold cross validation split is 92.29%. In the image segmentation task, 92.29% DSC means that the ROI drawn by our model is comparable to the manually drawn ROI. In Figure 5.2, we show the segmentation result of nnUnet and the result of our manual delineation. It can be seen that the two ROIs are basically the same.

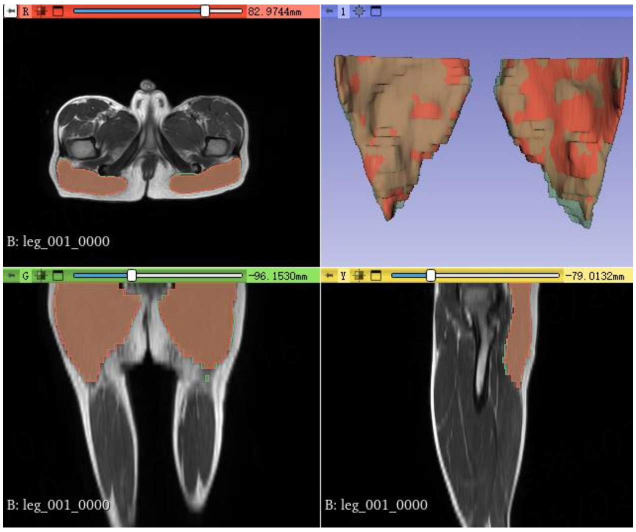

Figure 5.2 Segmentation results. The green ones are automatically segmented, and the red ones are manually outlined.

We believe that this segmentation result can replace the manual drawing of ROI. In addition, we use ICC to remove unstable radiomics, which also increases the reproducibility of our experiments. If you need the weights of our network, please contact the corresponding author.
